# Investigation on the Nanomechanics of Liposome Adsorption on Titanium Alloys: Temperature and Loading Effects

**DOI:** 10.3390/polym10040383

**Published:** 2018-04-01

**Authors:** Yiqin Duan, Yuhong Liu, Jinjin Li, Hongdong Wang, Shizhu Wen

**Affiliations:** State Key Laboratory of Tribology, Tsinghua University, Beijing 100084, China; duanyiqin91@163.com (Y.D.); sckler@163.com (H.W.); dpiwsz@mail.tsinghua.edu.cn (S.W.)

**Keywords:** liposome, nanomechanics, gel phase, titanium alloy, AFM

## Abstract

The mechanical properties of liposomes, determined by the lipid phase state at ambient temperature, have a close relationship with their physiological activities. Here, atomic force microscopy (AFM) was used to produce images and perform force measurements on titanium alloys at two adsorbed temperatures. The mechanical properties were evaluated under repeated loading and unloading, suggesting a better reversibility and resistance of gel phase liposomes. The liquid phase liposomes were irreversibly damaged during the first approach while the gel phase liposomes could bear more iterations, resulting from water flow reversibly going across the membranes. The statistical data offered strong evidence that the lipid membranes in the gel phase are robust enough to resist the tip penetration, mainly due to their orderly organization and strong hydrophobic interactions between lipid molecules. This work regarding the mechanical properties of liposomes with different phases provides guidance for future clinical applications, such as artificial joints.

## 1. Introduction

Phosphatidylcholine (PC) liposomes are widely used in various technologies [1,2,3,4,5,6], such as drug delivery systems in medicine, additives in cosmetics, gene delivery and food engineering, due to their biocompatibility. Recently, liposomes and bilayers have garnered increasing attention for their use as lubricants in joints [7,8,9,10]. Since 1989, bilayer structures have been always considered to be the primary lubricant in joints, but their bearing capacities were demonstrated to be only 1–2 MPa [11], less than the typical physiological pressure of 5 MPa (especially up to 20 MPa in some regions). Therefore, liposomes existing in vivo [12,13] were proposed to better explain articular lubrication. Low friction coefficients (from 2 × 10^−5^ to 0.01) can be obtained in certain liposome aqueous systems at a pressure of 10 MPa. On account of their highly hydrated PC head-groups and robust hydration shells [14,15], liposomes have been widely studied in different applications, such as surface force apparatuses and ex vivo cartilage devices [16,17]. Among these applications, the mechanical properties of the liposomes and bilayers are of critical importance. In particular, in lubrication application, their excellent mechanical performance can increase the bearing capacity of lubricants, thus reducing the wear of cartilage or implant surfaces. Several mechanical properties determine the stability of the bilayer and liposome, including the adsorbed structure on the substrate [18,19] and the fluidity or rigidity of the membrane [20,21], which in turn affect their tribology [8,22,23]. For example, 1,2-distearoyl-*sn*-glycero-3-phosphocholine (DSPC) liposome could greatly improve the lubrication performance due to its enhanced mechanical stability with longer PC acyl chains [8]. In addition, liposomes were reported to exhibit elastic behavior with lower friction [23], while bilayers were less robust with high friction for the reason that they were easily punctured by the atomic force microscopy (AFM) tip.

Micropipette aspiration [24,25] and other techniques [26,27,28,29] have often been used to measure the mechanical properties of giant liposomes (~μm diameter). For liposomes on the nanoscale, AFM has proved to be a precise instrument [30,31,32,33,34,35,36], providing images and detection of force variation during movement [37]. Typically, AFM has been widely used in nanomechanical studies by measuring Young’s modulus [30,31,36,38] or penetrating the lipid layer to detect its breakthrough forces [30,31,32]. The Young’s modulus of liposomes extracted from electric rays was first measured in 1997 with values of 0.2–1.3 MPa by adsorbing liposomes onto mica [38]. Then, Liang et al. probed small unilamellar egg PC vesicles on mica and measured a Young’s modulus of 1.97 MPa [30]. It was also found that the elastic modulus increased to 13 MPa with the addition of 50 mol % cholesterol [31]. Fery et al. measured the elasticity of 1,2-dipalmitoyl-*sn*-glycero-3-phosphatidylcholine (DPPC) at 110 MPa by using shell deformation theory [36]. Furthermore, the jump-in point from different bilayers attracted much attention when force-distance curves were measured [23,30,31,32]. This phenomenon can be considered to be bilayer penetration. For egg PC liposomes, two breaks were found during both the approach and retraction processes, corresponding to the opening and closing of vesicle bilayers [30]. 

Moreover, it is known that the saturation/unsaturation and length of the acyl chains directly determines the lipid phase transition temperature (*T*_m_) of phospholipids [8,32,33,37,39]. Therefore, the phase state of liposomes at ambient temperature could influence their mechanical properties as well. The results from an AFM morphology study [8] and measurements of Young’s modulus [32] from different PCs have shown that gel phase phospholipids are stiffer than those in the liquid phase. In most studies, two lipids in the liquid-disordered and gel-ordered phase at ambient temperature were used to investigate the effect of phase on the rigidity of membranes. However, the mechanical properties of two kinds of lipid membranes are related to not only the fluidity of lipid molecules, but also the acyl chain length and saturation [23]. For the same lipid, there is no difference in acyl chain length and saturation, so it is important to establish the relationship between phase state and the robustness of a lipid membrane. Furthermore, a few studies have reported studies on the effect of temperature on the mechanical performance of DPPC liposomes (*T*_m_ = 41 °C). Although the temperature in our body stabilizes at 37 °C, the local temperature between friction pairs, like joints, can reach a high value. Therefore, DPPC liposomes can exist either in the gel phase or in the liquid phase in the range of physiological temperatures. Moreover, it is known that the load applied on the knee changes a lot during a human’s usual activities, such as jumping or sitting. For further application, the repeated loading and unloading process is common in joints and will have a great influence on the bearing capacity and lubrication of joints. As a consequence, it is of great importance to evaluate mechanical properties under repeated loading and unloading and to investigate the effects of temperature on the nanomechanical properties of liposomes. In addition to these factors, the choice of substrate should not be underestimated. In this work, a polished titanium alloy, Ti6Al4V, was chosen as the substrate. It had been used widely in joint lubrication due to its excellent biocompatibility and mechanical properties [40,41]. In our previous work [18], liposomes adsorbed on titanium alloy were also found to have low friction, which provides knowledge relating to the improvement of the lubrication of artificial joints. Thus, it is necessary to expand the study on the mechanical behaviors of liposomes based on titanium alloy rather than mica [32] or silicon [8].

In the present study, liquid phase and gel phase DPPC liposomes were obtained by incubating them at target temperatures, which were above and below *T*_m_ respectively. The adsorption morphologies of liposomes were obtained by AFM. Then, the force measurements of liposomes were performed to measure the separation and critical force between the tip and the vesicle. Moreover, the mechanical properties of liposomes during repeated loading and unloading were characterized. Finally, the statistical data from all force-distance curves was summarized to better explain the bearing capacity of lipid membrane at the two phases.

## 2. Materials and Methods

### 2.1. Materials

1,2-dipalmitoyl-*sn*-glycero-3-phosphatidylcholine (DPPC) lipid was supplied by Avanti (Birmingham, AL, USA), with a *T*_m_ of 41 °C. Water used in sample preparation and measurement procedures was highly purified with a resistivity of 18.2 MΩ. Ti6Al4V foils (10 × 10 mm^2^, 1 mm thickness) were purchased from Goodfellow, Inc. (Oakdale, CA, USA) and were polished to achieve flat and smooth surfaces. The surface roughness was measured as 2.8 nm using a 3D white-light interfering profilometer (Nexview, Zygo, Middlefield, OH, USA).

### 2.2. Preparation of Liposomes and Adsorbed Surfaces

To form hydrated lipid sheets, lipid powders were dispersed into water and bathed for 1 h at a temperature above *T*_m_. Then, these hydrated lipid sheets were detached by oscillation to form self-closed multilamellar vesicles (MLVs) [7]. Next, the MLVs were downsized to form small unilamellar vesicles (SUVs, 20–200 nm) by stepwise extrusion through polycarbonate membranes (Whatman, Inc., Maidstone, Kent, UK), starting with 400 nm, then 200 nm, and ending with 100 nm, using an extruder (Avanti, Birmingham, AL, USA). The final size of the SUVs was 122.13 ± 35.25 nm, as determined by dynamic light scattering (DLS) using a zetasizer (Nano ZS, Malvern Panalytical, Malvern, UK). As with all procedures for extrusion, the experiments were performed at a temperature above *T*_m_.

Liposome-adsorbed surfaces were used during AFM. Polished Ti6Al4V surfaces were placed in a 0.1 mg/mL DPPC-SUV suspension for no less than 2 h. The incubation temperatures were chosen as 60 °C (*T*_1_) and room temperature (*T*_2_ = 25 °C). After incubation, the surfaces were rinsed by placing the adsorbed surfaces into a container of pure water, and shaken to remove excess, non-adsorbed liposomes.

### 2.3. Atomic Force Microscopy

Imaging of surfaces and nanomechanical measurements were performed using an Icon (Bruker, Santa Barbara, CA, USA). AFM Images were obtained in Peak Force Tapping mode in pure water. The peak force applied during imaging was 500 pN. Silicon nitride tips mounted on V-shaped cantilevers with a nominal spring constant of 0.35 N/m, length of 120 μm, width of 25 μm, and a nominal tip radius of 2 nm were used (SNL, Bruker, Santa Barbara, CA, USA). The force-distance curves were obtained after imaging using the same tip. To avoid tip contamination as a result of imaging, it is necessary to check that the tip is clean before each vesicle indentation (Figure S1). Before performing nanomechanical measurements, the deflection signal from the photodiode (*V*) was converted to force (*N*) by measuring the optical lever sensitivity, InvOLS (nm/V), and calibrating the spring constant with the equipartition theorem [42]. The force-distance curves were obtained by subtracting the cantilever deflection “*d*” from the vertical expansion of the piezo “*z*”, to which the cantilever was connected [43]. Thus, the separation between the tip and surface was calculated as “*z*-*d*”. The point of zero force was determined in the region where the separation was constant. The point of zero separation was determined at the onset where deflection was linear with the expansion of the piezo [30]. The duration of a single force measurement (both loading and unloading) was 10 s, and there was no interval between two force measurements. The approach or retraction velocity was 100 nm/s (hydrodynamics effect can be ignored).

## 3. Results and Discussion

### 3.1. Characterization of Liposomes Adsorbing on Surfaces

**Figure 1:**
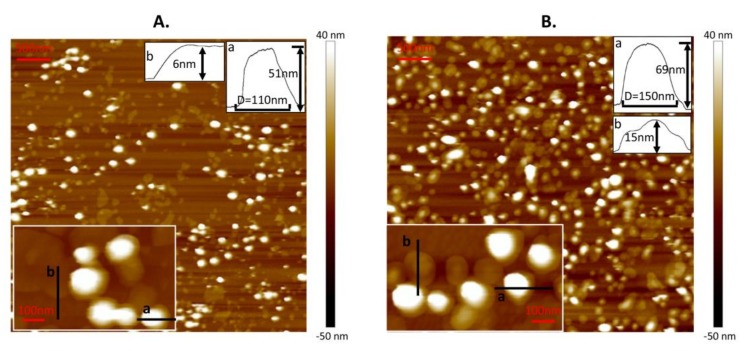
Atomic force microscopy (AFM) images of liposomes adsorbed onto Ti6Al4V at temperatures of (**A**) *T*_1_ = 60 °C and (**B**) *T*_2_ = 25 °C. Insets in the bottom left corner are magnified images. The lipid concentration is 0.1 mg/mL.

Before the nanomechanical measurements, DPPC liposomes were characterized by AFM to obtain their morphology on Ti6Al4V. In order to obtain individual vesicles dispersing on substrate following nanomechanical measurements, the lipid concentration was set as 0.1 mg/mL. The surface with adsorbed liposomes at a temperature above *T*_m_ is shown in Figure 1A. This coating is composed of spare vesicles and bilayers. Due to the adhesion effect from the substrate and the fusion induced between nearby vesicles at 60 °C [30], vesicles were easily ruptured to bilayers with a height of 6 nm (mark b). Meanwhile, for other intact vesicles (such as mark a), they were flattened with a height of 50.2 ± 13.4 nm and a diameter of 131.5 ± 20.3 nm (almost 183 vesicles were used to calculate the mean value of height and diameter). These values are lower than those of the unperturbed liposomes (calculated as 122.13 ± 35.25 nm from DLS) because of the adhesion force from the substrate and the compression from the tip during scanning. As a contrast, the surface with adsorbed liposomes at a temperature below *T*_m_ is shown in Figure 1B. This coating consisted of vesicles with two typical morphologies. Some of them were hemispherical (such as mark a) and others were spread like a “bump” [23] with a height no more than 20 nm (such as mark b). The height and diameter of hemispherical vesicles were 57.3 ± 12.1 nm and 128.6 ± 19.4 nm, respectively (almost 215 vesicles were used to calculate the mean value of height and diameter). The absence of bilayers in Figure 1B mainly results from the stability of the gel phase vesicle which makes fusion of the vesicles hard to achieve.

### 3.2. Three Different Structures of DPPC Vesicle Observed in a Continuous Loading and Unloading Process

**Figure 2:**
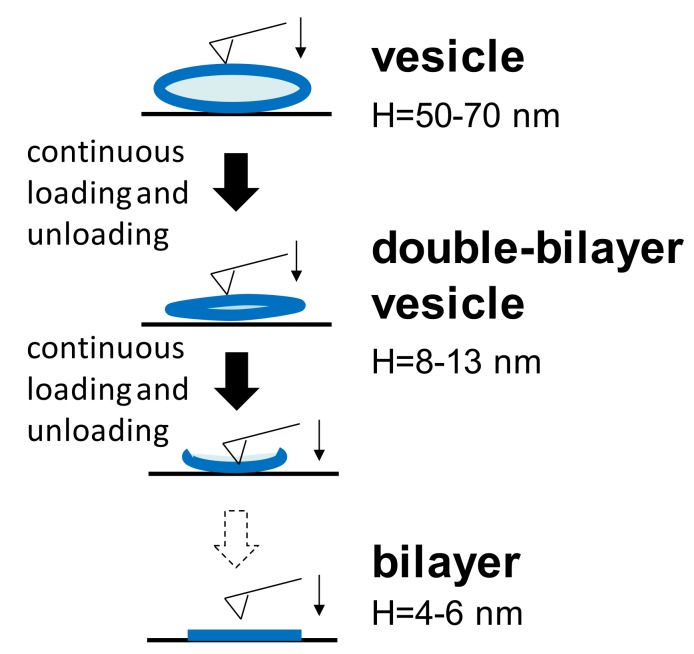
Three different structures appearing in continuous loading and unloading at two temperatures.

During nanomechanical measurements, a single force-distance curve (both approach and retraction) towards the vesicle adsorbed at either temperature was carried out. Furthermore, continuous loading and unloading was also performed on a single vesicle. It is common to find that, at either temperature, the spherical vesicle will change to another two different structures during the continuous loading and unloading process, as shown in Figure 2. Unperturbed vesicles are flattened on the substrate due to the adhesion between the vesicles and the substrate. In order to reduce the influence of the liposomes’ dimensions, the adsorbed vesicles used in all experiments had a narrow height of 50–70 nm. When the AFM tip continued to press this vesicle at certain iterations, the water content in the vesicle gradually reduced and the vesicle became more flattened with a typical height of 8–13 nm. Furthermore, the bilayer thicknesses in our measurements was 4–6 nm, corresponding to others’ results [23,30]. Therefore, a height of 8–13 nm is approximately equal to the thickness of the two bilayers, and thus, it is termed the double-bilayer vesicle. After further penetrations into the upper and lower bilayers by the tip, the double-bilayer vesicle ruptured and became a single bilayer. The bearing capacities of these different structures are discussed below.

### 3.3. Force Measurements of DPPC Vesicle Obtained at T_1_ = 60 °C

**Figure 3:**
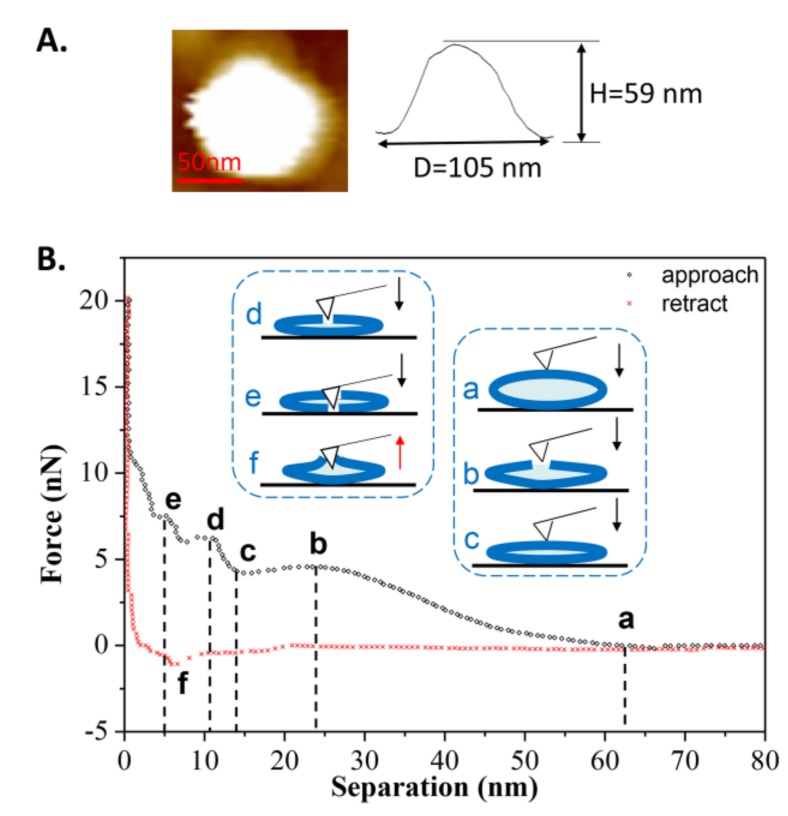
(**A**) AFM image of the vesicle prior to the first approach; (**B**) The force-distance curve and the schematic procedures describing the first tip approach and retraction on the DPPC vesicle obtained at *T*_1_ = 60 °C. The six states used in this whole article are described as follows: state a is the first contact when the tip approaches the spherical vesicle; state b is the appearance of a defect on the lipid membrane (water flows out of the defect); state c is the contact when the tip approaches close to the double-bilayer vesicle; state d is the tip penetration of the upper bilayer; state e is the tip penetration of the lower bilayer; and state f is the maximum adhesion during the retraction of the tip.

Figure 3B shows an example in which the tip approached a single vesicle for the first time at T_1_. During the approach process, a long-range repulsion starting at a separation of 62 nm appeared (state a), which is slightly larger than the height of the vesicle (measured as 59 nm in Figure 3A). The discrepancy was often 2–4 nm in this work, mainly due to the electrostatic force from the PC groups of the liposomes measured by AFM [37]. As the tip continued to approach, mechanical deformation of vesicle was observed. However, at a separation of 24 nm (state b), which is less than half of the vesicle’s height, a reduction in force appeared, indicating that there was a defect on the membrane through which water could flow out. Once the tip approached a separation of 13 nm (state c), consistent with the thickness of a double-bilayer vesicle, the normal force began to increase again, indicating that the tip compressed the vesicle again. When the remaining inner water of vesicle was completely squeezed out, the tip broke through the upper bilayer at a separation of 11 nm (state d). After that, the tip touched the lower bilayer and another jump occurred at a separation of 5 nm (state e). After these two jumps, the force increased rapidly due to the tip pressing against the hard surface. During the retraction, it exhibited a slight adhesion force at a separation of 6 nm (state f), indicating the bilayers were pulling the AFM tip when separating. The pulling process can last for a long distance until the tip has completely left the vesicles at a separation of 20 nm.

**Figure 4:**
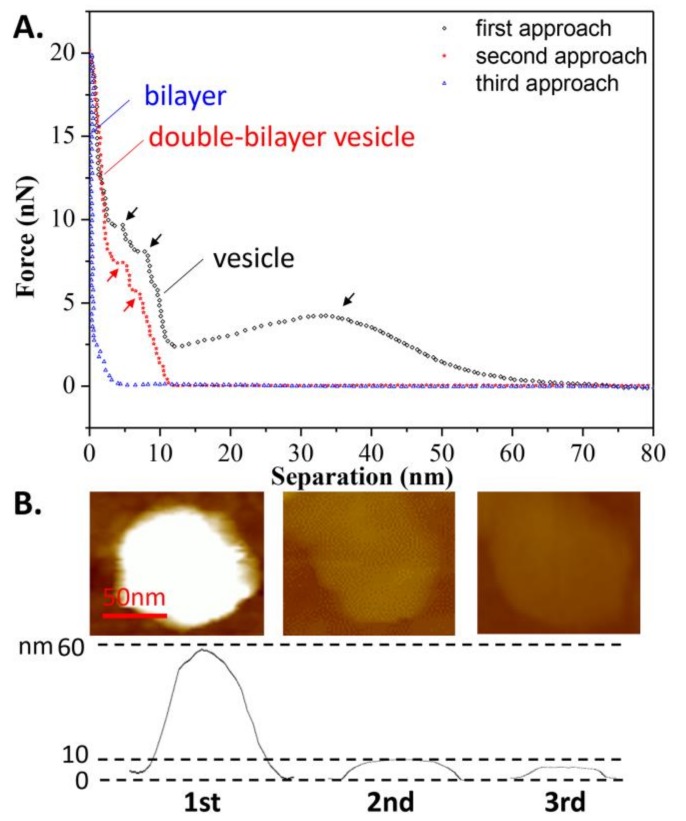
(**A**) Continuous approach curves on the DPPC vesicle obtained at *T*_1_ = 60 °C. Arrows refer to the jumps when the tip penetrates the bilayers; (**B**) AFM images of the vesicle prior to each approach.

In addition, the effects of continuous loading and unloading on the vesicle’s bearing capacity was also studied, as shown in Figure 4A. During the second tip approach, a repulsion force appeared at a separation of 12 nm, implying that after the first tip approach, the spherical vesicle became a double-bilayer vesicle. The 11-nm height of the vesicle, as measured prior to the second approach, verified this assumption, as shown in Figure 4B. Additionally, two jumps in the force-distance curve were observed at separations of 7 and 4 nm (marked in red arrows) during the second approach, corresponding to the breaking of the two bilayers. However, during the third approach, the repulsive force became apparent at a separation of 5 nm, indicating that the double-bilayer vesicle ruptured to form a bilayer after the second approach. This is consistent with the measured height of the bilayer from Figure 4B. Once the vesicle has become a bilayer, this process is irreversible. Since there is a defect on the membrane due to tip compression during the first approach, it can be inferred that the rest of the membrane rearranges and forms a new vesicle at state c, as shown in Figure 3 (mentioned as the double-bilayer vesicle). Due to the irreversible damage on the vesicle and its loss of interior water, the double-bilayer vesicle cannot recover back to its original height (~63 nm) before the second approach. Moreover, the rupture of the double-bilayer vesicle mainly arises from the weakness of newly formed upper bilayer, while the lower bilayer is firmly attached on the substrate without obvious damage.

### 3.4. Force Measurements of DPPC Vesicles Obtained at T_2_ = 25 °C

**Figure 5:**
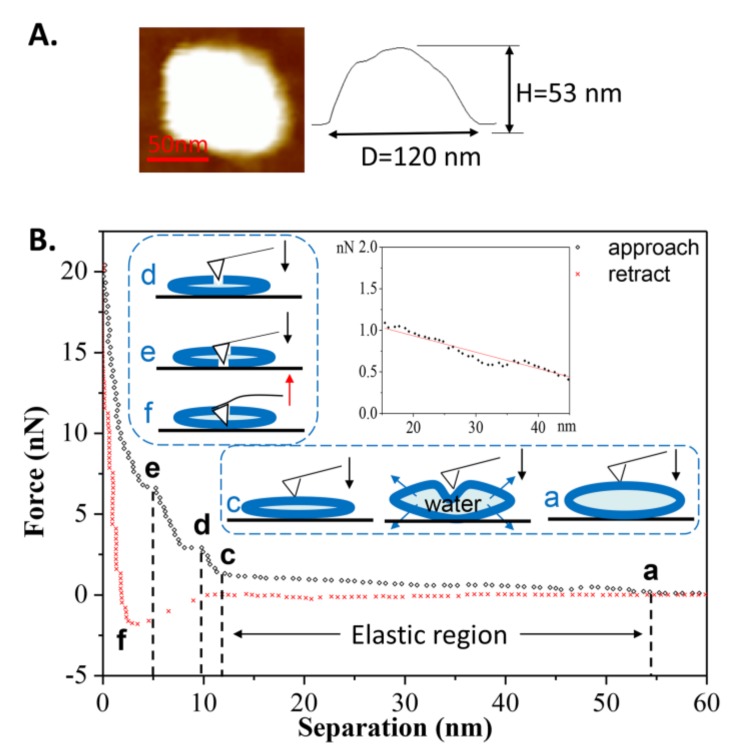
(**A**) AFM image of the vesicle prior to the first approach; (**B**) The force-distance curve and the schematic procedures describing the first tip approach and retraction on the DPPC vesicle were obtained at *T*_2_ = 25 °C. The inset shows the linear relationship between the normal force and separation (elastic region).

Another force-distance curve obtained at room temperature is shown in Figure 5B. During the first approach, a long-range repulsion began at a separation of 54 nm (state a), which is a bit larger than the height of the vesicle (measured at 53 nm in Figure 5A). The discrepancy was often 1–4 nm in this case. Compared to Figure 3B, no obvious defect is apparent in Figure 5B (without state b). On the contrary, the vesicle exhibited elasticity as observed from the linear deformation in the elastic region (magnified in the inset). Due to the flexibility and softness of the phospholipid membrane, the vesicle’s deformation caused some of the water inside the vesicle to be squeezed out from the lipid membrane, as illustrated in the schematic procedures. With the tip continuing to compress the double-bilayer vesicle (state c), two jumps occurred at separations of 9 and 5 nm (state d and state e), corresponding to the penetrations of the upper and lower bilayers. The same behaviors, from state c to state e, can also be observed in Figure 3. During the retraction, the maximum adhesion force occurred at a separation of 3 nm (state f) and then quickly reduced. The quick exit of the tip during the separation from 3 to 10 nm is a similar behavior to that of the tip leaving a hard substrate. Therefore, when tip is leaving the vesicle, the pulling process by bilayers cannot last long, resulting from the low fluidity [44] and low adhesion of the gel phase vesicles.

**Figure 6:**
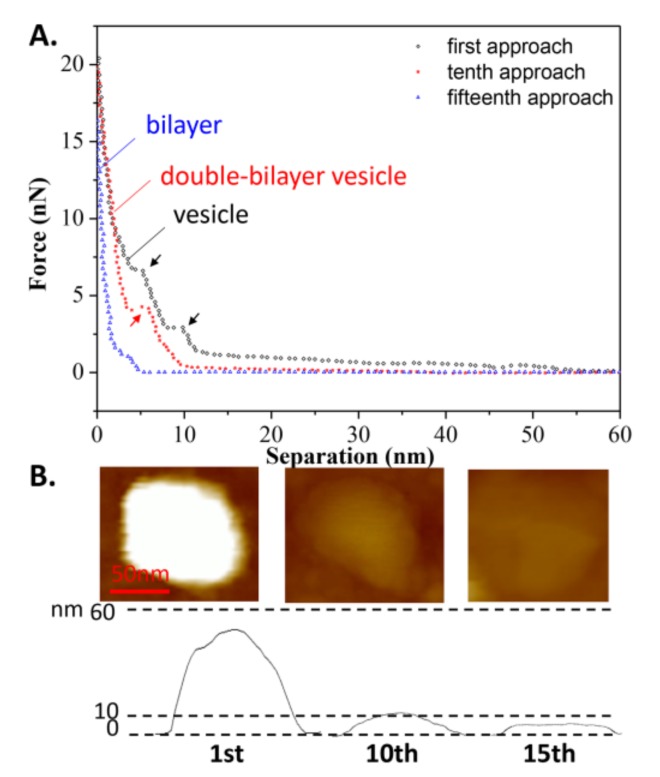
(**A**) The continuous approach curves of the DPPC vesicle obtained at *T*_2_ = 25 °C. The arrows refer to jumps occurring when the tip penetrates the bilayers; (**B**) AFM images before each approach.

The continuous approach curves obtained at *T*_2_ are also shown in Figure 6A for comparison with the curves in Figure 4A. It is surprising to see that the vesicle was able to withstand nine repeated iterations of loading and unloading, before becoming a double-bilayer vesicle during the tenth approach, with a height of 12 nm, as shown in Figure 6B (more AFM images are shown in Figure S2, indicating the vesicle was still intact before the tenth approach). This means that after each approach (less than ten iterations), the deformed vesicle began to absorb water again, and then recovered to its original shape (spherical vesicle). This reversible behavior was also found in the case of the double-bilayer vesicle. It was observed that the double-bilayer vesicle could withstand five repeated iterations of loading and unloading, before rupturing to a single bilayer during the fifteenth approach, with a height of 5 nm (Figure 6B).

**Figure 7:**
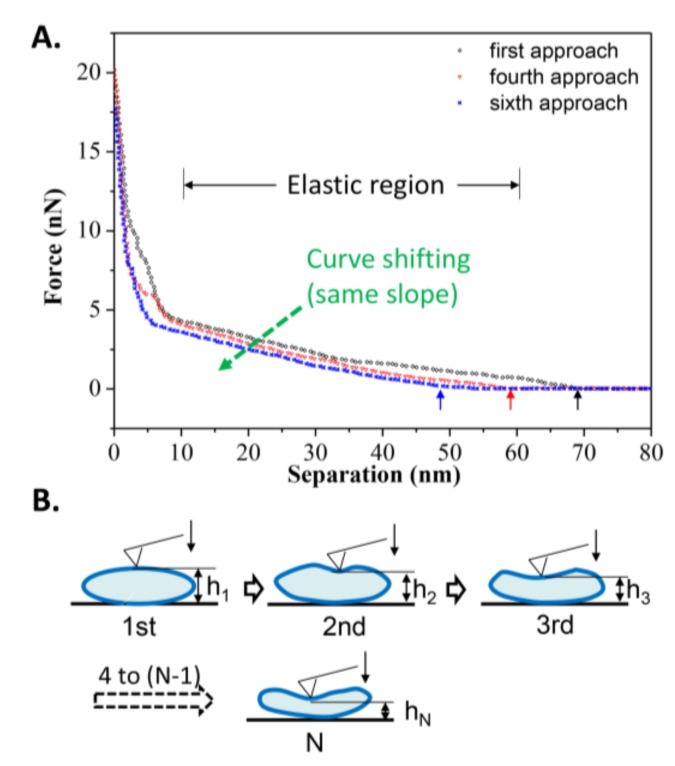
(**A**) Curve shifting of the repeated approach curves on an elastic DPPC vesicle obtained at *T*_2_ = 25 °C; (**B**) Schematic procedures simulating the repeated approach process. The arrows refer to the critical separation when repulsion force first appears.

Nevertheless, the number of iterations a gel phase vesicle can resist during repeated loading and unloading is limited, mostly due to hysteresis meaning that the vesicle cannot immediately recover to its original shape when the tip moves away from it, as observed in Figure 7A. Among these repeated approach curves, a shift of the elastic deformation region was observed (due to the same slope). It is obvious that the critical separation at which the repulsion force appears decreases with increasing loading iterations. From the first approach to the sixth approach, the critical separations were 69, 61, 60, 57, 50, and 48 nm, respectively. The average recovery time for vesicles can be inferred to be longer than the unloading time (~5 s), so the height of vesicle before the next approach decreases compared to the last approach, as illustrated in Figure 7B. Although a single vesicle is not able to resist more iterations of loading and unloading, the reversibility of the gel phase vesicle and the quick supplementation of new vesicles into the target area could maintain the excellent bearing properties of DPPC suspension during lubrication in joints.

By comparing Figure 3 and Figure 5, the crucial difference is whether the irreversible defect occurs in an elastic deformation region of the vesicle during the first approach. This difference would lead to different bearing capacities for vesicles, including the breakthrough force and the multiple iterations they can resist during repeated loading and unloading. Here, the statistics of five separations and corresponding critical forces among all spherical vesicles during the first approach are summarized in Table 1. At *T*_1_ = 60 °C, where the lipid molecules are in the liquid phase, the maximum statistical force for the last jump (state e) before the tip touches the hard surface is about 9.58 ± 2.91 nN, while the maximum force at *T*_2_ = 25 °C is 6.33 ± 2.85 nN. It seems that the vesicles in the gel phase are less robust than those in the liquid phase. However, the gel phase vesicles demonstrate a better healing capacity that can withstand repeated loading and unloading for more iterations (the normal force behavior keeps constant during each approach), as shown in Table 2.

Table 2 summarizes the normal bearing capacities during continuous loading and unloading for all structures of DPPC vesicles. At *T*_1_ = 60 °C, the spherical vesicles could only withstand repeated loading and unloading once, due to the destruction of the lipid membrane. The newly assembled vesicles were flat and weak, and they could only withstand up to two iterations before rupturing into single bilayers. The breakthrough force of the double-bilayer vesicles was 5.24 ± 2.25 nN, which is smaller than that of spherical vesicles, obtained as 9.58 ± 2.91 nN. This implies that the existence of water inside the vesicles contributes significantly to their bearing capacity. On the other hand, the breakthrough force of the single bilayers was reduced to 2.08 ± 0.82 nN, suggesting that the strength of the single bilayer was lower than that of the double-bilayer vesicle. At *T*_2_ = 25 °C, the spherical vesicles demonstrated excellent resistance to bearing loads up to 34 iterations of loading and unloading. Although the breakthrough forces of the double-bilayer vesicles at both temperatures were almost the same (the same result for bilayers), the double-bilayer vesicles at *T*_2_ = 25 °C were able to bear up to 12 iterations of loading and unloading.

### 3.5. Statistical Research on Comprehensive Bearing Capacity of DPPC Vesicles

**Figure 8:**
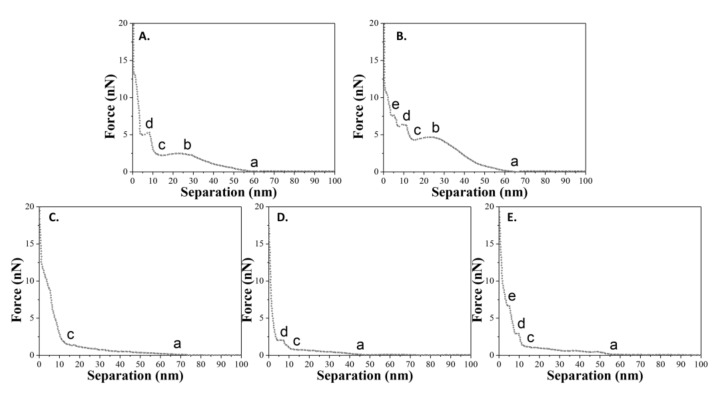
A summary of all types of force-distance curves (approach) for spherical vesicles adsorbed onto titanium alloy. (**A**,**B**) belong to the case where *T*_1_ = 60 °C; (**C**–**E**) belong to the case where *T*_2_ = 25 °C.

As mentioned previously, five states have been defined to describe the complete interactions between the tip and the sample during the approach process. The state a (or state c) refers to the contact of the tip and the vesicle (or double-bilayer vesicle). The state b refers to the irreversible defect only for liquid-phase vesicle. The state d and state e refer to the penetrations of upper and lower bilayers. However, it does not mean that the curve “abcde” for liquid-phase vesicles and the curve “acde” for gel-phase vesicles we mentioned before are representative for the samples (the maximal probability). In fact, the statistical analyses for all force-distance curves suggests that there are a variety of curves, and some curves only have two, three, or four out of the whole five states (states a to state e) for the vesicles, and one or two out of whole three states (states c to state e) for the double-bilayer vesicles. Figure 8 shows the summary of all types of force-distance curves for spherical vesicles during the approach process. For vesicles adsorbed at a temperature of *T*_1_, there are two types of curves. For vesicles adsorbed at a temperature of *T*_2_, there are three types of curves.

The probability of the appearance of these curves reveals an issue with the comprehensive bearing capacity of DPPC vesicles. Statistically, all types of force-distance curves for spherical and double-bilayer vesicles at two adsorbed temperatures are summarized in Table 3. For vesicles obtained at *T*_1_ = 60 °C (liquid phase lipid molecules), the probability of the appearance of curve “abcde” is maximal (62.5%), meaning that the tip can easily penetrate the vesicles and their two bilayers in most cases. Meanwhile, for double-bilayer vesicles, the probability of the appearance of curve “cde” is maximal (46.1%), indicating that both bilayers of the double-bilayer vesicle can be easily penetrated by the tip. In contrast, for vesicles obtained at *T*_2_ = 25 °C (gel phase lipid molecules), the probability of the appearance of curve “ac” is maximal for all cases (55.2%). This means that the tip cannot penetrate two bilayers (defined as states d and e above) in most cases, consistent with the robustness of vesicle assembled from the gel phase lipid molecules. In addition, for double-bilayer vesicles, the probability of the appearance of curve “c” is maximal (53.0%), meaning that the structure of the lipid molecules is strong enough that the double-bilayer vesicles can resist more than half of the tip penetrations. Despite this, the probability of the appearance of curve “cd” is 42.4%, indicating that some double-bilayer vesicles are penetrated by the tip (state d refers to the penetration of the upper bilayer). It is notable that the probability of the appearance for curve “acd” is lower (calculated as 38.6%) than that for curve “cd”. It is inferred that, compared to a spherical vesicle, there is not enough water between two bilayers to help the double-bilayer vesicle resist the tip penetration. Therefore, vesicles in the gel phase demonstrate a better bearing capacity, such that the tip cannot easily break their bilayers.

**Figure 9:**
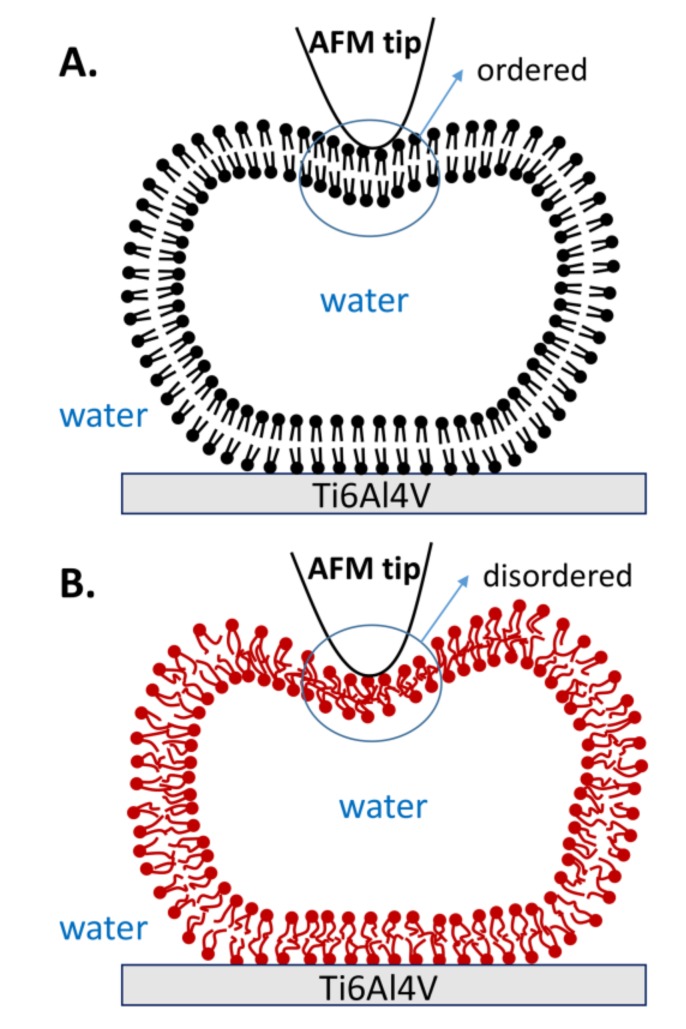
Schematic illustrations of vesicle deformation by AFM tip when lipid molecules are in (**A**) the gel phase and (**B**) the liquid phase.

The curves resulting from continuous repeated loading and unloading, combined with the obtained statistical data, provide strong evidence that the mechanical properties of vesicles are improved when lipid molecules are in the gel phase. The robustness of the gel phase vesicles is explained by the robustness and orderly organization of hydrophobic acyl chains, as shown in Figure 9A. Therefore, the lipid membrane is strong enough to resist most of the tip penetrations. Furthermore, the gel phase vesicles also exhibit excellent reversibility. Even if both bilayers are penetrated by the tip, the lipid molecules near the penetration area can quickly fill in the gap due to the strong interaction among the lipid molecules, and then they rearrange into an intact vesicle again. However, the acyl chains in the liquid phase are disordered, as shown in Figure 9B. Due to the reduced hydrophobic interactions between the liquid phase molecules, the stiffness of the membrane decreases [32]. Thus, the vesicle fails to resist the stretch of membrane when the tip compresses the vesicle with a large indentation, and a defect appears on the membrane (state b) in Figure 3. The defect causes irreparable damage on the upper lipid membrane, and the inner water flows out of the vesicle. Afterwards, the rest of the lipid membrane rearranges and forms a new vesicle with a smaller height, known as the double-bilayer vesicle. Due to the weakness of the lipid membrane formed in this short period of time, the double-bilayer vesicle is easily ruptured by tip penetration and finally becomes a single bilayer (Figure 4).

Moreover, the higher bearing capacity of the gel phase PC vesicles has been previously demonstrated [8,32,33,37,45]. In those studies, the gel phase DPPC membranes (*E* = 116 ± 45 MPa) were much stiffer than those of liquid phase 1,2-dioleoyl-*sn*-glycero-3-phosphocholine (DOPC) membranes (*E* = 13 ± 9 MPa) at 20 °C [32]. Meanwhile, the increase in acyl chain length was also found to increase the liposomes’ structural integrity on the substrate surface when studied by AFM, which has been considered the primary reason why gel phase DPPC liposomes (C16) have lower friction coefficients than liquid phase DMPC liposomes (C14) at room temperature [8]. However, the influences of acyl chain length and saturation [23] on mechanical properties from the two kinds of lipids cannot be ignored. Leonenko et al. [45] demonstrated that the breakthrough force for DPPC bilayers decreases with increased temperature (*T*_m_ was included). In this work, repeated loading and unloading experiments based on DPPC lipids were conducted, suggesting a reversibility and an enhanced resistance of DPPC vesicles under the gel phase. The nanomechanical study of DPPC vesicles adsorbing on titanium alloys in this work provides evidence that the gel phase vesicles are more robust and can withstand more iterations of loading and unloading, thereby possibly improving their mechanical and lubrication performance in clinical applications, such as artificial joints.

## 4. Conclusions

In this work, DPPC liposomes were adsorbed onto titanium alloys at room temperature and 60 °C, in gel and liquid phases. The gel phase liposomes showed better mechanical properties, bearing up to 34 iterations of loading and unloading without irreversible damage of vesicles. Even double-bilayer vesicles in the gel phase were able to withstand up to 12 iterations. However, liposomes in the liquid phase were easily damaged in the elastic region during the first approach and reassembled to form flattened vesicles. The double-bilayer vesicles were also weak and ruptured into bilayers quickly. The weakness of the liquid phase vesicles and double-bilayer vesicles results from the disorderly arrange of acyl chains and the reduced hydrophobic interactions between lipid molecules. Statistical data also provides strong evidence that the lipid membranes in the gel phase are not easily penetrated by tips, with a penetration probability of less than 50% among all curves. This work provides a fresh perspective to evaluate the mechanical properties of liposomes under repeated loading and unloading and discuss the effect of lipid phase on liposomes’ bearing capacity. Both of these factors are necessary in joint lubrication.

## Figures and Tables

**Table 1:** Summary of tip-surface distance and corresponding force for the first tip approach on DPPC vesicles under two adsorbed temperatures. H is the height of the original vesicle. The statistics of the curves at *T*_1_ and *T*_2_ come from 20 samples (vesicles), respectively.

*T*_1_ = 60 °C > *T*_m_			*T*_2_ = 25 °C < *T*_m_		
State	Distance/nm	Force/nN	State	Distance/nm	Force/nN
a	≈H	0	a	≈H	0
b	≤1/2H	2.93 ± 1.05	-	-	-
c	11.89 ± 1.15	2.53 ± 0.87	c	12.07 ± 1.12	1.42 ± 0.83
d	8.93 ± 0.98	6.88 ± 2.50	d	8.48 ± 0.89	3.23 ± 1.80
e	5.28 ± 0.68	9.58 ± 2.91	e	5.02 ± 0.83	6.33 ± 2.85

**Table 2:** Summary of bearing capacity on continuous loading and unloading for three structures of DPPC vesicles at two adsorbed temperatures. “Frequencies” is the maximal number of repeated loading and unloading iterations where vesicles (or double-bilayer vesicles) can return to their original shape after each approach. “Force” is the maximum breakthrough force for each structure (bilayer is not included). The statistics of curves at *T*_1_ and *T*_2_ come from 20 samples (vesicles), respectively.

	Structure	Vesicle	Double-Bilayer Vesicle	Bilayer
*T*_1_ = 60 °C > *T*_m_	Frequencies	1	1–2	-
	Force/nN	9.58 ± 2.91	5.24 ± 2.25	2.08 ± 0.82
*T*_2_ = 25 °C < *T*_m_	Frequencies	3–34	4–12	-
	Force/nN	6.33 ± 2.85	4.86 ± 2.11	2.19 ± 0.47

**Table 3:** Statistical summary of all types of force-distance curves (approach) and corresponding probabilities of appearance for spherical vesicles and double-bilayer vesicles. For each temperature, the data comes from 120 samples (vesicles) which have similar sizes.

	Curves of Vesicle	Probability of Appearance	Curves of Double-Bilayer Vesicle	Probability of Appearance
*T*_1_ = 60 °C > *T*_m_	abc	0	c	30.8%
	abcd	37.5%	cd	23.1%
	abcde	62.5%	cde	46.1%
*T*_2_ = 25 °C < *T*_m_	ac	55.2%	c	53.0%
	acd	38.6%	cd	42.4%
	acde	6.2%	cde	4.6%

## Supplementary Materials

The following are available online at http://www.mdpi.com/2073-4360/10/4/383/s1, Figure S1: A typical clean substrate curve before vesicle indentation, Figure S2: AFM images of vesicle between iteration number 2 and number 9 during continuous loading and unloading at *T*_2_ = 25 °C.
