# Supplementary material for: Investigation on the Nanomechanics of Liposome Adsorption on Titanium Alloys: Temperature and Loading Effects
Source: Polymers (Basel). 2018 Apr 1;10(4):383. doi: 10.3390/polym10040383 (PMC6415199; doi:10.3390/polym10040383)
Supplement: Supplementary file 1 [file polymers-10-00383-s001.docx]

**Supplementary Materials**

Investigation on the Nanomechanics of Liposome Adsorption on Titanium Alloys: Temperature and Loading Effects

Yiqin Duan, Yuhong Liu *, Jinjin Li *, Hongdong Wang and Shizhu Wen

State Key Laboratory of Tribology, Tsinghua University, Beijing 100084, China; [duanyiqin91@163.com](mailto:duanyiqin91@163.com) (Y.D.); [sckler@163.com](mailto:sckler@163.com) (H.W.); [dpiwsz@mail.tsinghua.edu.cn](mailto:dpiwsz@mail.tsinghua.edu.cn) (S.W.)

***** Correspondence: [liuyuhong@tsinghua.edu.cn](mailto:liuyuhong@tsinghua.edu.cn) (Y.L.); [lijinjin@mail.tsinghua.edu.cn](mailto:lijinjin@mail.tsinghua.edu.cn) (J.L.);
Tel.: +86-10-6278-8387 (Y.L.); +86-10-6278-9482 (J.L.)

1. A Typical Clean Substrate Curve before Vesicle Indentation

Before vesicle indentations, the force curves on the Ti6Al4V substrate were obtained to check whether the tip was clean. Figure S1 shows an example of a typical clean substrate curve in our experiments. With the clean tip, the force curves on 1,2-dipalmitoyl-*sn*-glycero-3-phosphatidylcholine (DPPC) vesicles could be carried out.

|  |
| --- |

**Figure S1.** A typical clean substrate curve before vesicle indentation.

2. Atomic force microscopy (AFM) Images of Vesicle between Iteration Number 2 and Number 9 during Continuous Loading and Unloading at *T*_2_ = 25 °C

As a supplementary figure for Figure 6, Figure S2 shows a few AFM images before each approach (from the second approach to the ninth approach). These images demonstrate that the vesicle is still intact. Meanwhile, the height of the vesicle is decreasing slowly with increased iterations, which agrees well with the results in Figure 7.

|  |
| --- |

**Figure S2.** AFM images of the vesicle between iteration number 2 and 9 during continuous loading and unloading at *T*_2_ = 25 °C.

© 2018 by the authors. Submitted for possible open access publication under the
terms and conditions of the Creative Commons Attribution (CC BY) license (http://creativecommons.org/licenses/by/4.0/).
